# Girls’ transition from participation in a modified sport program to club sport competition - a study of longitudinal patterns and correlates

**DOI:** 10.1186/s12889-018-5609-0

**Published:** 2018-06-08

**Authors:** Rochelle Eime, Jack Harvey, Melanie Charity

**Affiliations:** 1 Faculty of Health, Federation University Australia, Ballarat, Australia; 2 Institute for Health and Sport, Victoria University, Melbourne, Australia

**Keywords:** Sport, Modified sport programs, Female

## Abstract

**Background:**

Participation in sport is very popular for young children. Many children participate in entry-level modified sports programs. These programs are modified to match the developmental capacity of children and are aimed at development of fundamental motor skills and sport-specific skills, rather than competition. There is limited research on the longitudinal tracking of children in these programs and into club-based competition. Research suggests that most children drop-out of the sport and do not transition into club-based competition. Furthermore, more females than males drop-out of sport. The aim of this study is to investigate longitudinally, the patterns and demographic predictors of children’s transition from modified sport programs to club sport competition for females.

**Methods:**

This study analysed sport participation for females in a popular Australian, predominantly female, sport. Players of the modified sports program were followed over 4 years to determine their pattern of transition: transition to junior player status, withdraw from the sport, or continue in the modified program. Pattern of transition was compared across age (4–10), geographical region (metropolitan/non-metropolitan) and socio-economic status (SES). Logistic regression was used to model the effect of the three factors on the likelihood of transition.

**Results:**

A total of 13,760 female children (aged 4–10) participated in the modified sport in the first year. The majority (59%) transitioned from the modified sport program and into club competition. However the rate of transition varied with age, residential location and socio-economic status, and there was an interaction between region and SES, with SES having a significant influence on transition in the metropolitan region. The peak sport entry age with the highest rates of transition was 7–9 years.

**Conclusions:**

This study demonstrated that whilst the majority of female participants continued participantion and tranisitioned from the modified sport program and into club competition, the strongest correlate of transition was age of entry, with transition rate peaking among those who commenced at age 7–9 years. It is recommended that, in order to maximise continued participation, sport policy and strategic developments should consider the possibility that targeting the very young is not the optimum recruitment strategy for fostering continued sport participation.

## Background

Sport is a popular leisure-time physical activity, especially for children [1, 2]. In the UK recent research reports that 72% of children aged 9 and 63% of those aged 12 participate in a sport at a community sports club [1]. In a recent Australian study of 520,102 sport participants aged 4–100 the highest rates of participation were within the 10–14 followed by 5–9 year age groups, and nearly one third of sport participants were aged 10–14 years (28%) [2].

Participation in sport has been associated with higher rates of meeting the physical activity guidelines compared to those not participating in sport [3–5]. Club-based participation in sport contributes considerably to leisure-time physical activity at health enhancing levels [4, 6]. Furthermore, there are reports that primary school children participating in organised sport are less sedentary and perform more moderate to vigorous physical activity [4] than non-participants. Children who participate in sport are also more likely to participate in physical activity during adolescence [1] and into adulthood [7, 8]. In addition, sport participation among children and adolescents is more strongly linked to improved psychological and social health than are other individual based leisure-time physical activities [9, 10]. More specifically, regular participation in club-based sport for children can improve motor coordination levels [11]. Because of these positive health and developmental outcomes, sport participation has been specifically recommended as a potential strategy to increase physical activity levels, and promote physical fitness in children and youth [12, 13].

Whilst participation in sport is popular for children, there is extensive research showing that participation dramatically decreases during adolescence [2, 9, 14, 15]. One study reported that from a peak through ages 10–14 (28% of all participants), participation declined dramatically during ages 15–19 years (15% of all participants) [2]. In another study of a total of 465,403 sport participants, it was reported that participation rate per resident population in Victoria, Australia was 40% for ages 10–14 and 23% for 15–19 years [14].

Much of the sport participation literature concerning levels, trends and determinants relates to older children and adolescents [16–19], with less research on young children. A systematic review of dropout from organised sport for children and youth found that most studies focused solely on adolescents, and not young children [20]**.** The studies of determinants have implications for the drop-out evident through late adolescence [21], but are not necessarily associated with factors of sport drop-out in sport entry level ages for very young children.

A recent longitudinal study of young sport adopters (aged 4–12) tracked 209,336 children over a 4-year period [22]. These children were participating in a modified sport program, and the research tracked whether or not they transitioned into club based participation/competition [22]. Modified sports programs for young children are designed among other things, to develop fundamental motor skills and sport-specific skills for future participation [23], and the rules and equipment are modified from the traditional ‘adult’ version of the sport [22]. The fundamental focus is on participation and development of skills, rather than competition [22]. This study found that the great majority of children withdrew from participation in the sport during the 4-year period rather than transitioning from the modified sport program to club competition [22]. Across the ages 4–12, 24.5% of females and 13.6% of males transitioned to club sport competition within the 4-year period. Furthermore, two-thirds of children (67.4%) withdrew from participation in their sport after the first year/season of the study [22]. This study indicates that the issue of drop-out in sport may also be a problem in early childhood, and not only a major factor amongst adolescents. Modified sports are entry level programs; sports organisations (clubs and governing bodies) should have an interest in retaining players, which requires these young people to transition into club based competition. Furthermore, from a health perspective continued participation in sport and physical activity is important. Whilst there is literature on the intrapersonal and interpersonal factors relating to sport participation, and consistent findings regarding barriers such as lack of enjoyment, perceptions of competence, social pressures and competing priorities [20, 21], we know little about the factors affecting very young sport adopters.

In addition to understanding the trends, it is important to investigate the determinants or predictors of transition and drop-out. We know that sport participation rates are much lower for females than males [2, 9, 14, 24]. Initial descriptive research on tracking of modified sport participation through to club-competition has shown that there are differences across age at baseline for both males and females [22], however there has been no statistical modelling of the effects on transition of age, or other factors such as socio-economic status (SES) and geographical region. Further to this, SES is a likely predictor in this cohort as there us much evidence that sports club participation is associated with SES at an area level [1, 25] and at a household level [19]. In general higher levels of SES or financial support are associated with higher levels of sport participation [1, 19, 26]. However, another recent study of sport and physical activity reported no differences in participation rates across neighbourhood levels of SES [3]. Another study of adults reported that the highest and lowest SES group were less likely to participate in sport than the middle SES group [24]. In terms of regional effect, participation in sport is often higher in regional and rural areas than in metropolitan regions [3, 14].

The aim of this study was to investigate longitudinally, the patterns and demographic predictors (age, SES and region) of female children’s transition from modified sport programs to club sport competition.

## Methods

Data for this study were collected as part of the Sport and Recreation Spatial project (www.sportandrecreationspatial.com.au) and have been previously described in detail [2, 14]. This study drew participants from a female-dominated club-based team sport in the Australian state of Victoria between 2012 and 2016. This sport was ranked within the top 10 organised physical activities and regular club-based physical activities in Australia [27].

The base year for this study was 2013. Participants in the modified sports program in 2013 were divided into two groups: (1) those who were also participants in 2012, and (2) those who were not. For this study, because age of commencement in the modified program was a key focus, the former group was excluded. The latter group (“2013 commencers”) were followed over 4 years (2013–2016), with their pattern of participation being categorised as one of: transition to junior competition player status, withdrawal from the sport, or continuation in the modified program. The pattern of participation was first compared descriptively across age (4–10 years of age in single years) and geographical region (metropolitan/non-metropolitan) groups in the base year (2012). Socio-economic status (SES) was represented by tertiles of the Socio-Economic Indexes for Areas (SEIFA) Index of Relative Socio-Economic Advantage and Disadvantage scores for the postcode of residence (1 = most disadvantaged to 3 = most advantaged).

Transition was defined as being registered as a player of the modified sport in the base year and then as a junior competition player in any of the subsequent years. The non-transition group included those who withdrew from the sport, those who remained in the modified program, those who had a break of one or more years then returned, and players who returned to the modified program after one or more year of junior competition participation.

Logistic regression was used to model the effect of the factors age at commencement (in single years), geographical region (metropolitan/non metropolitan) and socio-economic status (SES) (SEIFA tertiles) on the likelihood of transition. The model including main effects and interactions between each pair of factors showed a significant interaction between geographical region and SEIFA tertile. To explore this relationship further, a composite “region / SEIFA tertile” variable was calculated. The final model included age and the composite region / SEIFA tertile variable.

## Results

Table 1 shows that a total of 13,760 female children commenced participation in the modified sports program in the base year (2013). Consistently across both regions, most players were aged 7–9. While numbers in metropolitan and non-metropolitan regions were quite similar, a higher proportion of players in the metropolitan region resided in areas within the highest SEIFA tertile than was the case in non-metropolitan areas. Over half of the metropolitan players (54%) resided within the highest tertile of SEIFA areas, compared to only 8% of non-metropolitan players. Conversely, over half of the non-metropolitan participants resided within the lowest tertile SEIFA areas (52%), compared only 12% of metropolitan participants.

**Table 1:** Female Participant Characteristics

	Metropolitan n	Non-metropolitan n	Victoria n
Age in 2013 (years)			
4	133	265	398
5	537	961	1498
6	1072	972	2044
7	1670	1360	3030
8	1619	1891	3510
9	1659	1400	3059
10	85	136	221
SEIFA IRSAD Tertile			
Tertile 1	780	3660	4440
Tertile 2	2338	2795	5133
Tertile 3	3657	532	4189
Transition pattern			
Transition	4486	3623	8109
Withdraw	2113	2856	4969
Continue	176	508	684
Total	6775	6985	13,760

The majority of female (59%) transitioned from the modified sport program and into junior club competition. However, there were regional differences. Of participants in the metropolitan region 66% transitioned, compared to 52% of those in the non-metropolitan region. However, more participants within non-metropolitan regions continued participation compared to metropolitan participants. Furthermore, a higher proportion of non-metropolitan participants withdrew (40%) compared to 31% of metropolitan participants.

**Fig. 1:**
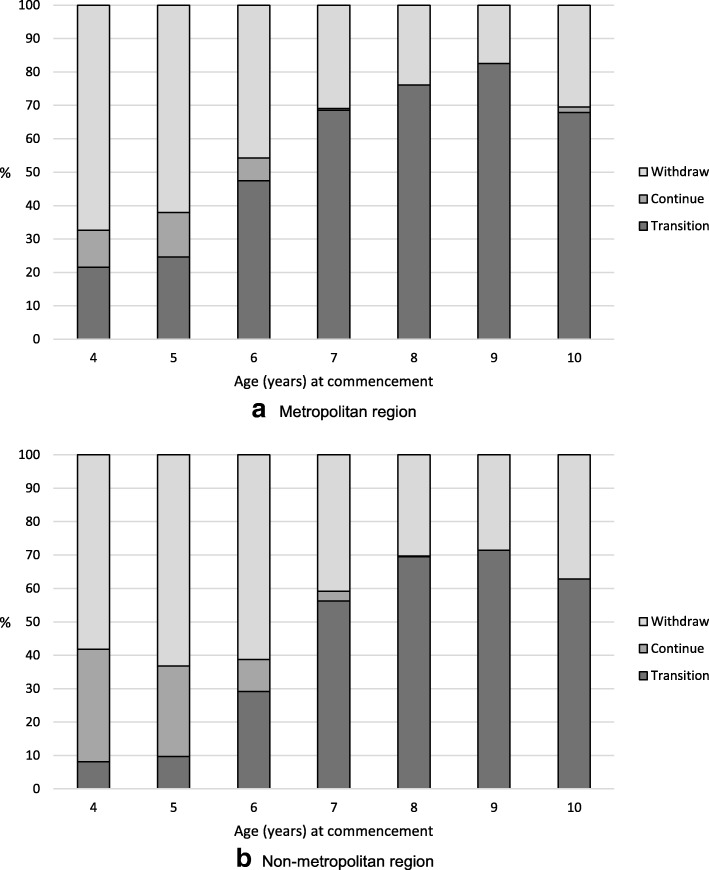
Transition pattern of female registered players. **a**. Metropolitan region. **b**. Non-metropolitan region

Transition patterns (transition, continue, withdraw) by age in years at commencement (4–10) and region (metroplolitan and non-metropolitan) are shown in Fig. 1. The overall transition pattern was similar for both the metropolitan and non-metropolitan regions, however there was a considerably higher proportion of transition among the youngest metropolitan commencers aged 4–7 years. The rates of transitioning for older commencers (aged 8–10 years) was quite similar across both regions, and peaking at age 9 for both regions (83% metropolitan and 71% non-metropolitan). The highest withdraw rates were amongst the youngest commencers (aged 4–5 years), and was highest amongst 4 year old metropolitan participants (67%) and 5 year old non-metropolitan participants (63%). Not surprisingly, the highest rates of continued participation were amongst the younger commencers and especially higher in non-metropolitan compared to metropolitan participants.

Results from the final logistic regression model are shown in Table 2. Those who commenced at age 6 and above were significantly more likely to transition than 4 year-olds. The likelihood of transitioning generally increased from age 6 to a peak at age 9, before declining at age 10.

**Table 2:** Results of logistic regression analysis of female participants

			Logistic regression analysis			
		n	OR	95% CI		*p*-value
Age	4	437	1.00			
	5	1735	1.09	0.73	1.63	0.659
	6	2423	3.26	2.24	4.75	< 0.001
	7	3563	7.88	5.44	11.40	< 0.001
	8	3969	12.97	8.96	18.77	< 0.001
	9	3385	15.97	11.01	23.17	< 0.001
	10	247	10.35	6.35	16.89	< 0.001
Geographical region & SEIFA tertile^a^	Metropolitan & Tertile 1	905	1.00			
	Metropolitan & Tertile 2	2597	1.82	1.50	2.22	< 0.001
	Metropolitan & Tertile 3	4133	2.30	1.91	2.78	< 0.001
	Non-metropolitan & Tertile 1	4225	1.07	0.89	1.29	0.483
	Non-metropolitan & Tertile 2	3277	1.15	0.95	1.41	0.157
	Non-metropolitan & Tertile 3	622	0.98	0.75	1.29	0.912

^**a**^Because of a statistically significant interaction between geographical region and SEIFA tertile, these two variables were restructured as a single composite variable

SES had a significant effect on the likelihood of transitioning in metropolitan areas, but not in non-metropolitan areas. Those living in metropolitan areas with higher SEIFA values were significantly more likely to transition than those in the lowest tertile. The likelihood of transitioning increased with each SEIFA tertile. In non-metropolitan areas, likelihood of transitioning was similar, in all three SEIFA tertiles, to that of the lowest metropolitan tertile.

## Discussion

This study builds upon previous research by investigating the demographic correlates of sports participation transition from modified sports to club competition [22]. It demonstrates that, for the particular female-dominated sport studied, the likelihood of transition from modified sport to club competition was affected by age, region of residence, and SES of residential location.

A positive, in terms of continued sport participation, is that the majority of participants did transition in the sport. Overall 59% transitioned which is much higher than a previous report where less than 25% of females transitioned within a 4-year period [22]. The higher rates of transition in this study may relate to it being a predominantly female sport whereas the previous research was across multiple sports, and some of these were traditionally male sports [22]. In another Australian study of sport trajectories from age 5, 10, 15 and 17, 48% of the females were consistent sport participants, 34% were sport drop-outs and 18% were non sport participants [9]. However this study related to participation in any organised sport outside of school.

Whilst in the present study the majority of participants transitioned, this pattern was more likely to occur amongst metropolitan participants (66%) compared to non-metropolitan participants (52%). Recent research has shown that overall sports participation in an Australian context is considerably higher in non-metropolitan compared to metropolitan regions [28]. It was conjectured that this relates to the significant social role that community sport plays in non-metropolitan regions and that sports offerings in non-metropolitan areas are predominantly the traditional few, rather than the vast choice of leisure activities which are available within metropolitan areas [28]. However the present study shows that in non-metropolitan areas, regardless of SES tertile, the likelihood of transitioning from modified sports to club competition is as low as in the lowest metropolitan SES tertile. Perhaps there are fewer opportunities for the large numbers of modified sports club participants to move into one or only a few teams in non-metropolitan regions compared to greater opportunities in the higher SES metropolitan regions. This requires further investigation.

It was also found that a considerably higher proportion of non-metropolitan participants resided within the lowest tertile of SEIFA (52%) compared to only 12% of metropolitan participants. Furthermore, over half of the metropolitan participants were in the highest SEIFA tertile (54%) compared to only 8% of those non-metropolitan participants. This may in part be due to the fact that non-metropolitan areas in general have lower SES than metropolitan areas [28], but it is consistent with other research where lower SES has been reported as a consistent and strong correlate of both participation in sport and time spent in sport [12]. Each lower category of neighbourhood SES was associated with lower odds of sport participation [12]. In a study of children aged 7–12 years, sports club participation was significantly associated with SES, with fewer children from poorer areas playing sportr [1, 19]. More specifically high SES children participated about an hour per week more in sports compared with low SES children [25].

Continued sport participation across childhood may be too expensive for those in the most disadvantaged socio-economic areas. Furthermore, it has been recognised that the actual transition from modified sport to club-competition is associated with increased participation costs such as club memberships, uniforms and other associated travel costs to home and away games [22]. It may be that the modified sports programs provide a value-for-money sporting opportunity, but then club competition becomes too costly for families with very low SES. Furthermore, children’s participation in sport and different types of sports may be influenced by the family SES, with some sports being more expensive to participate in than others.

The peak sport entry age, with the highest rates of transition, was 7–9 years. This may be the optimal entry level age for the female participants to transition (at some stage over a 4-year period) into club-based sport. It would seem that a person starting to play sport at age 10 or older may be limited in their competency compared to those who have played for several years [22], and perceived competency and motor control for children is a significant predictor of involvement in sport and physical activity more generally [11, 29]. Furthermore, this current study demonstrates that if female children start playing organised sport at a very early age, this may hinder transitioning from the modified form to club competition in that particular sport. It has been suggested that many of the very young children are not going to be motivated to participate in the same modified sport program for 4–5 years before they are eligible to play club competition at around the age of 8 years [22]. They are likely to become bored and either sample another sport or drop out of sport all together [22]. The reasons for children starting to play organised sport at such an early age of 4 or 5 needs to be investigated. Is this a parental push, or is it driven by sport policy encouraging an increase in the number of participants and leading to the targeting younger age groups, with associated heavy sport and corporate marketing? Is starting too young to play organised sport an actual hindrance to their sport involvement later in childhood? Both sports organisations and parents need to consider the entry age that children should be entering into formal organised sports programs.

## Conclusions

In conclusion, this study has demonstrated that whilst the majority of female participants continued participantion and tranisitioned from this particular modified sport program and into club competition, there were differences in the rate of transition associated with age of commencement, residential location and socio-economic status. The strongest correlate of transition was age of entry into the modified program, with transition rate peaking among those who commenced at age 7–9 years. It is recommended that, in order to maximise continued participation, sport policy and strategic developments should consider the possibility that targeting the very young is not the optimum recruitment strategy for fostering continued participation in sport.

## Abbreviations

SEIFA: Socio-Economic Indexes for Areas
SES: Socio-economic status
